# A model for cell migration in non-isotropic fibrin networks with an application to pancreatic tumor islets

**DOI:** 10.1007/s10237-017-0966-7

**Published:** 2017-10-09

**Authors:** Jiao Chen, Daphne Weihs, Fred J. Vermolen

**Affiliations:** 1 Delft Institute of Applied Mathematics, Delft University of Technology, Delft, The Netherlands; 2 Faculty of Biomedical Engineering, Technion-Israel Institute of Technology, 3200003 Haifa, Israel

**Keywords:** Cell migration, Cell-based model, Semi-stochastic model, Stromal extracellular matrix, Pancreatic tumor islet

## Abstract

Cell migration, known as an orchestrated movement of cells, is crucially important for wound healing, tumor growth, immune response as well as other biomedical processes. This paper presents a cell-based model to describe cell migration in non-isotropic fibrin networks around pancreatic tumor islets. This migration is determined by the mechanical strain energy density as well as cytokines-driven chemotaxis. Cell displacement is modeled by solving a large system of ordinary stochastic differential equations where the stochastic parts result from random walk. The stochastic differential equations are solved by the use of the classical Euler–Maruyama method. In this paper, the influence of anisotropic stromal extracellular matrix in pancreatic tumor islets on T-lymphocytes migration in different immune systems is investigated. As a result, tumor peripheral stromal extracellular matrix impedes the immune response of T-lymphocytes through changing direction of their migration.

**Electronic supplementary material:**

The online version of this article (doi:10.1007/s10237-017-0966-7) contains supplementary material, which is available to authorized users.

## Introduction

Cell migration is a directed movement of cells which typically includes amoeboid and mesenchymal movement. Cell migration is driven by (combinations of) several mechanisms: chemical cues (chemotaxis or haptotaxis where the cues are in the fluid phase or extracellular matrix, respectively), mechanical cues (mechanotaxis, being tensotaxis or durotaxis, respectively, migration according to mechanical tensions and movement up to a stiffness gradient), electrical cues (electrotaxis), by light activation and by random walk. Cell migration is an integral part of many different biomedical processes, such as wound healing, organ development, tumor growth and cancer metastasis. Moreover, it is critical in the framework of the immune system responses which are indispensable for clearing the body from hazardous chemicals, pathogens and mutated cells, such as cancer cells. Therefore, understanding cell migration is crucially important for finding ways to improve therapies.

The immune response is essential for all living organisms. In antitumor immune responses, tumor-specific T cells, in particular CD8$^{+}$ T cells, play an indispensable role. However, some cancer cells are able to escape the engulfment by T cells through various mechanisms. One possibility could be that the tumors build stromal barriers against immune cells. Pancreatic ductal adenocarcinoma (PDAC) is known for its profuse desmoplastic stroma which is composed of activated fibroblasts, collagen and extracellular matrix (Rhim et al. 2014). The desmoplastic stroma plays an important role in the tumor progression; however, its function is likely to be dynamic over time since its cellular and noncellular constituents change over time (Angeli et al. 2009; Özdemir et al. 2014). In the literature, there is some controversy whether stromal constituents support or inhibit tumor progression. Rhim et al. (2014) demonstrate that at least some stromal constituents can act to physically restrain rather than promote tumor progression. Whereas, according to Salmon and Donnadieu (2012), the stroma may support tumor progression by preventing the immune system from reaching and destroying the tumor. They observe that tumor islets (T-islets), which are surrounded by rich stromal networks, can form a major obstacle for T cells-mediated antitumor activities. Moreover, Hanahan and Weinberg (2011) state that some stromal cells cause immune suppression and hence promote tumor survival and growth. Therefore, the effects of the tumor microenvironment, and specifically the stroma, on tumor progression are still unclear. This could lay the foundation for improvement of cancer therapy.

Much experimental work has been done on cancer and cancer cells. However, the tumor microenvironment and its immune mechanisms have only recently become an important focus, and thus, the availability of experimental data and results are still limited. Therefore, there is an urgent need to strengthen multidisciplinary tumor research including input from medicine, biology, engineering and mathematics. Developing new insights into tumor behavior and response in connection with its environment as well as immune system interations requires a strong link between available experimental results and the development of updated hypotheses. In order to facilitate this link and to be able to forecast tumor behavior under various experimental circumstances that lie beyond the currently available experimental results, a quantification of the hypotheses into mathematical relations is indispensable. Mathematical models can be developed on several scales ranging from continuum-based macro-models to cell-based micro-models. Since cell-based models often use measurable experiment-based quantities (like cell migration rates, etc.), they are very attractive though their implementation over large domains is possibly expensive. Since in the present study, we are interested in the small spatial length scale of the order of millimeters, the model that we currently work with is cell-based. Cell-based models can be divided into several classes:

- Lattice-based models, which include cellular automata, lattice gas cellular automata and cellular Potts models, which are described in the review (Van Liedekerke et al. 2015). In cellular automata, cells may occupy a single lattice site; herewith, one is able to simulate a large population of cells (Block et al. 2007; Radszuweit et al. 2009). In comparison, lattice gas cellular automata models include velocity channels next to their positions (Rothman and Zaleski 2004). Furthermore, the cellular Potts models are characterized by energy functionals that determine the probability of a change of state at a lattice point. More information can be found in the works by Merks et al. (2009), Van Oers et al. (2014) and the pioneering work by Glazier and Graner (1993);
- Particle models are formalisms where each cell is treated as an individual particle with a fixed geometry (circles or spheres in the two- and three-dimensional cases) and where the cells are allowed to migrate throughout the region according to several chemical, mechanical or electrical signals. For an overview, we refer the interested reader to consult (Vermolen 2015), while for specific implementations, we refer to Kim et al. (2015) and Ribeiro et al. (2017) in the modeling of filopodia. In particular, Ribeiro et al. (2017) report on how the filopodia contribute to cell migration. Furthermore, Drasdo and Hoehme (2005), Byrne and Drasdo (2009), Vermolen and Gefen (2012, 2013b) and Vermolen et al. (2015) elaborate on the context of the immune system to fight cancer;
- Cell-shape evolving models are representations where the geometry of the cell changes during its migration. This migration may result from various signals. Here, one should mention the approach by Madzvamuse and George (2013), which deals with the migration and movement of the cells with a viscoelastic inner cell structure, the model by Borau et al. (2014), which is based on a voxel approach, as well as the approach by Vermolen and Gefen (2013a) which is based on a division of the cell surface into meshpoints that are connected to each other and to the center of the cell. Vermolen et al. (2014) extend the approach to a multicell and multi-physics environment to simulate the immune system.
- Hybrid discrete-continuum models, which are feasible models for large multicellular systems (Van Liedekerke et al. 2015). This class, based on treating cells as individual entities and other signals through continuum-scale approaches, is explained further in Kim et al. (2007). Furthermore, the hybrid approach has been used for simulation of would healing (Yang et al. 2013) and angiogenesis (Milde et al. 2008), etc.

This manuscript will focus on the simulation of cell migration in T cells-mediated antitumor response with an application to pancreatic tumors. The work by Salmon and Donnadieu (2012) has shown that in many cases pancreatic carcinoma consist of T-islets which are surrounded by stromal regions where collagen is oriented parallel to the circumference of the T-islet. Since the T cells migrate faster in the direction of the orientation of the collagen fibers, the T cells merely circle around the T-islets and thereby hardly enter them. Hence, the T cells are unable to function in neutralizing the cancer cells. To be able to identify ways to change these circumstances, we have developed mathematical models that reproduce the experimental phenomenon as much as possible and allow to simply and rapidly develop and test hypotheses on the process mechanisms and predict experimental outcomes. Using the simulations, we propose and evaluate a possible therapy, based on injecting or stimulating isolated endothelial cells that are not connected to the blood vessel network and letting them invade the tumor islet through the collagen network around them. The idea is to exploit the endothelial cells ability to degrade the network such that the T cells are able to invade the T-islet and be able to interact with and neutralize the cancer cells. This idea has not been implemented as a therapy to fight cancer; however, our aim is to simulate this process to show its potential applicability.

In this manuscript, we aim at a microscale phenomenological description that the T cells migrate in the vicinity of the T-islet. We will extend the formalism by Vermolen and Gefen (2012) to non-isotropic fibrin networks where the formalism by Cumming et al. (2010) will be used and extended such that we can model geometrically evolving cells in non-isotropic environments. Next to the T cells, we will take into account the migration, proliferation, apoptosis of all other cells as well as mutation of benign epithelial cells.

The paper is organized as follows: first, the mathematical formalism is introduced, and then the numerical method is presented. This is followed by the numerical simulations where the model and its results will also be discussed. Finally, we draw some conclusions on the simulation results and give some directions for future research.

## Mathematical model

The model addresses several biological processes, which will allow simulation of immune response to a tumor in different microenvironments. Specifically, we include the migration, division, apoptosis, mutation of cells, the chemical signaling as well as the immune reaction in a non-isotropic environment. In addition, the contractile forces exerted by cells are accounted for by a simplified mechanical balance. To this extent, a domain of computation $\varOmega \subset \mathbb {R}^2$ is introduced where $\varOmega _\mathrm{T} \subset \varOmega$ denotes the T-islet. The islet $\varOmega _\mathrm{T}$ is surrounded by a stromal layer, which contains a high-density fibrin network with orientation parallel to the circumference of $\varOmega _\mathrm{T}$; this subdomain is denoted by $\varOmega _\mathrm{F} \subset \varOmega$ and it does not overlap with $\varOmega _\mathrm{T}$.

To encode a mathematical model, the following procedures and assumptions are used in the development of the present formalism: (1) to keep the computations short in CPU time, we consider a two-dimensional domain of computation; (2) all cells are hemispherical and the projection onto the two-dimensional substrate is a circle; (3) each cell has two discrete states: viable or dead; (4) each viable epithelial cell exerts a traction force and is able to migrate or proliferate; (5) cells that collide repel each other by the contact forces that they exert in the normal direction. In the following subsections, we provide the formalisms for each cell condition.

### The migration of the epithelial cells

Traction forces are crucial for adhesion and migration of cells and affect the intercellular communication and as well as for, among others, shape maintenance and mechanical signal generation, see the experimental studies in Wang and Lin (2007) and Reinhart-King et al. (2008). For the sake of completeness of the model description in this manuscript, we present the cell migration model that was developed in Vermolen and Gefen (2012). The model formulation for cell migration was based on the experimental observations by Reinhart-King et al. (2008). Firstly, we consider the distant communication of cells through traction force. Later, we will deal with the repulsive force that is induced by physical contact. Tensile forces are applied by cells to their microenvironment using the actomyosin machinery. Cells generate tensile forces internally as myosin motors induce lateral, relative motion of two actin filaments. An actin filament may connect to the microenvironment through transmembrane integrins. The external part of the integrin may then connect to the substrate or extracellular matrix, thus transmitting the intracellular force (Massalha and Weihs 2017). Slight deformation of the substrate caused by a stress gives a strain energy *U*, which reads as:

1 $$\begin{aligned} U = \frac{1}{2}V \sigma \varepsilon = \frac{1}{2}VE \varepsilon ^2 = \frac{1}{2} \frac{V}{E} \sigma ^2, \end{aligned}$$

where *V* denotes the deformation volume, $\sigma$ denotes stress, $\varepsilon$ denotes strain of the substrate at the center of cell and *E* is the Young’s modulus from Hooke’s law, given by

2 $$\begin{aligned} E = \frac{\sigma }{\varepsilon }. \end{aligned}$$

We use $M_{i}^{0}$ to represent the strain energy density, that is the energy per unit of volume, which follows from the exertion force $F_{i}$ at the position of cell *i*. Then the strain energy density is dictated by

3 $$\begin{aligned} \begin{aligned}&M_{i}^{0} = \frac{1}{2}\sigma \varepsilon = \frac{1}{2}E_\mathrm{s}( \mathbf{r}_{i})\varepsilon ^2 = \frac{1}{2}\frac{\sigma ^2}{E_\mathrm{s}( \mathbf{r}_{i})},&\\&\mathrm{for} \ i \in \{1,\ldots ,n\}, \end{aligned} \end{aligned}$$

where $E_\mathrm{s}(\mathbf{r}_{i})$ represents the local elastic modulus of the corresponding substrate. Furthermore, we neglect compressibility of the extracellular matrix. This is motivated by the experimentally observed Poisson ratio of 0.48 (Massalha and Weihs 2017; Kristal-Muscal et al. 2015). The above relation is able to handle the non-uniformity of the substrate stiffness. Further, $\mathbf{r}_{i}$ denotes the position of cell *i*. If we use *L* and *d* for the thickness and vertical displacement of the deformed substrate, then $\varepsilon$ is given by

4 $$\begin{aligned} \varepsilon = \frac{d}{L}, \end{aligned}$$

and hence the strain energy density can be calculated by

5 $$\begin{aligned} M_{i}^{0} = \frac{1}{2}E_\mathrm{s}(\mathbf{r}_{i})\left( \frac{d}{L}\right) ^2, \quad \mathrm{for} \ i \in \{1,\ldots ,n\}. \end{aligned}$$

Hooke’s Law is used for a low strain by

6 $$\begin{aligned} \varepsilon = \frac{1}{E_\mathrm{s}(\mathbf{r}_{i})} \frac{F_{i}}{\pi R^2}, \quad \mathrm{for} \ i \in \{1,\ldots ,n\}. \end{aligned}$$

From the above equation and Hooke’s Law, we get

7 $$\begin{aligned} M_{i}^{0} = \frac{1}{2\pi ^2}\frac{F_{i}^2}{E_\mathrm{s}(\mathbf{r}_{i})R^4}, \quad \mathrm{for} \ i \in \{1,\ldots ,n\}, \end{aligned}$$

where *R* represents the cell radius. The finding by Merkel et al. (2007) shows that the strain energy density decays exponentially approximately with the decay factor given by

8 $$\begin{aligned} \lambda _{i}=\frac{E_\mathrm{s}(\mathbf {r}_{i})}{E_\mathrm{c}}, \quad \mathrm{for} \ i \in \{1,\ldots ,n\}. \end{aligned}$$

Here, $\lambda _{i}$ is used to represent the signal attenuation ratio of elasticity modulus of substrate $E_\mathrm{s}(\mathbf{r}_{i})$ and elasticity modulus of cell $E_\mathrm{c}$. We calculate the strain energy density $M_{i} (\mathbf{r})$ due to the cell position $\mathbf r$ with center position $\mathbf{r}_{i}$ by

9 $$\begin{aligned}&M_{i}(\mathbf{r})= M_{i}^{0}\mathrm{exp}\left\{ -\lambda _{i} \frac{\parallel \mathbf{r}- \mathbf{r}_{i}\parallel }{R}\right\} ,\nonumber \\&\quad \mathrm{for} \ i \in \{1,\ldots ,n\}. \end{aligned}$$

As outlined in Vermolen and Gefen (2012), the energy density is a scalar number; hence, it can be summed to obtain a total strain energy density $M(\mathbf{r})$ due to all cells at position **r** as follows,

10 $$\begin{aligned} \begin{aligned}&M(\mathbf{r})= \sum _{j =1}^{n} M_{j}(\mathbf{r}) = \sum _{j=1}^{n} M_{j}^{0}\mathrm{exp}\left\{ -\lambda _{j} \frac{\parallel \mathbf{r}- \mathbf{r}_{j}\parallel }{R}\right\} ,\\&\quad \mathrm{for} \ j\in \{1,\ldots ,n\}. \end{aligned} \end{aligned}$$

Thence for cell *i* at time *t*, its own sensed mechanical stimulus $M(\mathbf{r}_{i})$ is represented by

11 $$\begin{aligned} M(\mathbf{r}_{i})&=\sum _{j = 1}^{n}M_{j}(\mathbf{r}_{i}) = \sum _{j=1}^{n}M_{j}^{0}\mathrm{exp}\left\{ -\lambda _{j} \frac{\parallel \mathbf{r}_{i}-\mathbf{r}_{j}\parallel }{R}\right\} \nonumber \\ {}&=M_{i}^{0}+\sum _{j=1_{j \ne i}}^{n}M_{j}^{0}\mathrm{exp}\left\{ -\lambda _{j} \frac{\parallel \mathbf{r}_{i}-\mathbf{r}_{j}\parallel }{R}\right\} ,\nonumber \\&\mathrm{for} \ i, j \in \{1,\ldots ,n\}. \end{aligned}$$

where $\mathbf{r}_{i}$ and $\mathbf{r}_{j}$ denote the position of cell *i* and cell *j*, respectively. According to Vermolen and Gefen (2012), the displacement direction of a cell is a linear combination of all the unit vectors between this cell *i* and others caused by their mechanical signals. For cell *i* and cell *j*, the unit vector is $\mathbf{e}_{ij} = \frac{\mathbf{r}_{i}-\mathbf{r}_{j}}{\parallel \mathbf{r}_{i}-\mathbf{r}_{j}\parallel }$, and the total displacement of cell *i* during a time step $\Delta t$ is parallel to

12 $$\begin{aligned} \mathbf{z}_{i}=\sum _{j=1_{j\ne i}}^{n}M_{j}(\mathbf{r}_{i}(t))\mathbf{e}_{ij}, \quad \mathrm{for} \quad i, j \in \{1,\ldots ,n\}, \end{aligned}$$

where $\mathbf{r}_{i} (t)$ is the cell *i* position at time *t*, and $\mathbf{z}_{i}$ is a vector to guide the direction of cell movement and hence the corresponding total unit vector is $\hat{ \mathbf{z}}_{i}=\frac{\mathbf{z}_{i}}{\parallel \mathbf{z}_{i}\parallel }$. Taking the mechanical stimulus into consideration, total displacement over a time is calculated by

13 $$\begin{aligned} d\mathbf{r}_{i}(t)=\alpha _{i} M(\mathbf{r}_{i}(t))\hat{\mathbf{z}}_{i}\mathrm{d}t, \quad \mathrm{for} \ i \in \{1,\ldots ,n\}, \end{aligned}$$

where $\alpha _{i}$ is a parameter with dimension $\left[ \frac{\mathrm{m}^3}{\mathrm{Ns}}\right]$ and the shear force is directed along the substrate, which acts perpendicularly to the exertion force. For viable cells, Gefen (2010) achieves an expression for $\alpha _{i}$

14 $$\begin{aligned} \alpha _{i}= \frac{\beta _{i}R^3}{\mu F_{i}}, \quad \mathrm{for} \ i \in \{1,\ldots ,n\}, \end{aligned}$$

where $\beta _{i}$ quantifies the mobility of the portion of the cell surface that is in physical contact with the substrate of a viable cell and $\mu$ is the cell substrate friction coefficient, which equals 0.2 according to Gefen (2010). Viable cells move according to the mechanical stimulus that they sense; however, they are also observed to move (partly) according to random walk and hence magnitude of movement should be revised to

15 $$\begin{aligned}&d\mathbf{r}_{i}(t)=\alpha _{i}M(\mathbf{r}_{i}(t))\hat{\mathbf{z}}_{i}\mathrm{d}t+\sqrt{2D} \mathrm{d}{\mathbf{W}(t)}, \nonumber \\&\mathrm{for} \ i \in \{1,\ldots ,n\}, \end{aligned}$$

where $\mathrm{d}{} \mathbf{W}(t)$ is a vector Wiener process and *D* is cell diffusivity.

Epithelial cells move under the influence of strain energy as well as random walk in the circle islet. The *detection threshold* $\epsilon$ is introduced as a minimum strain energy signal for remote cells to detect each other. Therefore, the total signal strength a cell senses should satisfy

16 $$\begin{aligned}&M_i(\mathbf{r}) = M_i^0\mathrm{exp}\left\{ -\lambda _i\frac{\parallel \mathbf{r}-\mathbf{r}_i\parallel }{R}\right\} \ge \epsilon ,\nonumber \\&\mathrm{for} \ i \in \{1,\ldots ,n\}. \end{aligned}$$

Reinhart-King et al. (2008) found that the largest distance for a cell to detect is around $\hat{d} =$ 30 $\upmu$m with different elasticity moduli of substrate (approximately 5 kPa) and cell (approximately 0.5 kPa). This distance may depend on the phenotype of the cell (Sen et al. 2009). Hence, the threshold $\epsilon$ is defined by

17 $$\begin{aligned}&\epsilon = M_i^0\mathrm{exp}\left\{ -\lambda _i\frac{\hat{d}}{R}\right\} \approx 1.99 \times 10^{-54}, \nonumber \\&\quad \mathrm{for} \ i \in \{1,\ldots ,n\}. \end{aligned}$$

Here $\epsilon = 0$ kg $\cdot \,\upmu$m/min$^2$ is used taking the rounding error of the computer into account. Once the cells come into physical contact with each other, the force reacting against invagination pushes the cells away from one another. This is treated in the next subsection.

### The repulsion of the contacting cells

Cells will not occupy the same space under normal circumstances. However, cells can have direct mechanical and physical contact with their neighbors, which is associated with shape changes in general. In this model, cells are allowed to migrate toward each other and to prevent them from occupying too much common space, a repulsive force is added to our model with cells that remain circular at all times.

Gefen (2010) introduces a repulsive invagination force into the cell contact force, which is also incorporated in the computational framework. The elastically impinging cells will generate a repulsive force to repel each other, which is determined by the invagination distance and contact radius. This invagination force will translate to the concept of energy through the computation of the amount of work. This has been worked out in Vermolen and Gefen (2012). Then, the strain energy density as a result of intercellular contact between cell *i* and cell *j* is given by

18 $$\begin{aligned} M^{ij}=\frac{4}{15 \sqrt{2}}\frac{E_\mathrm{c}}{\pi }\left( \frac{h}{R}\right) ^{\frac{5}{2}},\quad \mathrm{for} \ i, j\in \{1,\ldots ,n\}, \end{aligned}$$

where $M^{ij}$ and *h*, respectively, denote the strain energy density produced by the elastic interaction and indentation distance between the two neighboring cells. We calculate *h* by

19 $$\begin{aligned} h = \mathrm{max}(2R-\parallel \mathbf{r}_{ij}\parallel , 0 ), \quad \mathrm{for} \ i, j\in \{1,\ldots ,n\}, \end{aligned}$$

where the $\mathbf{r}_{ij}$ represents the distance between cell *i* and cell *j*, and total strain energy density $\hat{M}_{i}$ between cell *i* and cell *j* by

20 $$\begin{aligned} \hat{M}_{i}=M(\mathbf{r}_{i})-M^{ij},\quad \mathrm{for} \ i, j\in \{1,\ldots ,n\}. \end{aligned}$$

We phenomenologically assume that the repulsive motion is proportional to the strain energy density that the cell experiences. Note that this phenomenological treatment does not incorporate Newton‘s Law. Note that the migration of the cells contains two components. The first component follows from long-distance communication. The second component, which only sets in if $$h > 0$$, results from repulsive motion due to physical contact between the cells. Having two cells, this will imply that an equilibrium is reached if $$\hat{M}_i = 0$$. This results into an equilibrium distance between the positions of the cells. This also means that the cells mechanically touch over a certain area, and herewith one can phenomenologically consider this as a measure of cell–cell adhesion. In the case of multiple cells that are in mechanical contact, the $$ M^{ij}$$ term has to be summed over all the cells that are in mechanical contact with cell *i*. Imagine that cell *i* is in mechanical contact with cells $$\{i_1,\ldots ,i_k\} \subseteq \{1,\ldots ,n\}$$, then the above equation is written as,

21 $$\begin{aligned} \hat{M}_i(\mathbf{r}) = M(\mathbf{r}_{i})-M_i^{mc}(\mathbf{r}_i), \quad \mathrm{for} \ i \in \{1,\ldots ,n\}, \end{aligned}$$

where $M_i^\mathrm{mc}(\mathbf{r}_i)= \sum _{j \in \{i_1,\ldots ,i_k\}}M^{ij}$, which is the mechanical contact term of the strain energy density. Note that the repulsive forces can be balanced with attracting forces, and hence, the cells can partly overlap and be in physical contact. Therewith, the model allows treatment of collective cell migration.

### The division, apoptosis and mutation of the cells

Each cell has a life cycle that affects its ability to migrate and is characterized by the following stages: (1) G1, increase of RNA and ribosome during this phase the cell does not move actively; (2) S, synthesis of DNA. Furthermore, the cell is mobile during this phase; (3) G2, synthesis of RNA and protein. During this phase, the cell volume increases and the cell is mobile; (4) M, cell mitosis and during this phase the cell does not move actively. We will incorporate this cell proliferation process in our simulation in the future. We model cell division, apoptosis as well as mutation fully using stochastic principles. Using the same principles given in Vermolen et al. (2015) and Vermolen (2015), we assume that the probability of cell division, apoptosis or mutation obeys a simple memoryless exponential distribution and that it is only affected by the total strain energy density a cell endures, which is given by $f_{t_n}({\lambda }, t)\Delta t$ during the interval $(t_{n}, t_{n}+\Delta t)$. Here, ${\lambda }$ $({\lambda } > 0)$ is the probability per unit of time (here per minute) of cell division, apoptosis or mutation after $t_n$, and $f_{t_n}({\lambda }, t)$ is defined as,

22 $$\begin{aligned} f_{t_{n}}({\lambda },t)={\lambda }\ \mathrm{exp}(-{\lambda }(t-t_{n})), \end{aligned}$$

and hence,

23 $$\begin{aligned} P(t\in (t_n, t_n+\Delta t))&= \int _{t_n}^{t_n+\Delta t}f_{t_n}({\lambda }, t)\mathrm{d}t\nonumber \\&= 1-\mathrm{exp}(-{\lambda }\Delta t). \end{aligned}$$

Note that if ${\lambda }\Delta t \ll 1$, then

24 $$\begin{aligned} P(t \in (t_n, t_n+\Delta t)) = {\lambda }\Delta t + O({\lambda }\Delta t)^2, \end{aligned}$$

where *O* is Landau order-symbol to describe the limiting behavior of a function.

To realize it in the code, we let the system randomly generate a number $\xi \sim u[0,1]$ taken from an uniform distribution. The cell, respectively, divides, dies or mutates if and only if

25 $$\begin{aligned} 0 \leqslant \xi \leqslant 1-\mathrm{exp}(-{\lambda }\Delta t), \end{aligned}$$

where, as mentioned earlier, $\lambda$ stands for the probability rate parameter for cell division, apoptosis or mutation.

In this model, cell proliferation, apoptosis as well as mutation happen under the premise of satisfying two kinds of conditions:

- Firstly, we simulate cell proliferation, apoptosis as well as mutation using the probability rates $\lambda _\mathrm{d}$, $\lambda _\mathrm{a}$ and $\lambda _\mathrm{m}$, respectively, which depend on the total strain energy density that the cell senses as a result of physical contact with its neighbors. We hypothesize that when a cell in a monolayer is in mechanical contact with six cells in 2D then it reaches a steady state. By Eq. (21), we calculate the value of $\hat{M}_i(\mathbf{r}) = M(\mathbf{r}_i)-M_i^{mc}(\mathbf{r}_i)$ that corresponding with a cell being surrounded and just being in physical contact with six other cells such that the cell boundaries of each pair of cells coincide at one point has a value of approximately 0.0125 kg $\cdot \,\upmu$m/min$^2$. We find that the equilibrium value of the strain energy density for one cell in contact with one other cell is approximately 0.03 kg $\cdot \, \upmu$m/min$^2$ (see, Fig. 2). Herewith, we assume that one epithelial cell has sufficient space to divide if $\parallel \hat{M}_i(\mathbf{r}) \parallel<$ 0.03 kg $\cdot \,\upmu$m/min$^2$ and in the same way, a cancer cell can divide if $\parallel \hat{M}_i(\mathbf{r}) \parallel < 0.04$ kg $\cdot \,\upmu$m/min$^2$. Furthermore, a cell is able to mutate or die with a bigger $\parallel \hat{M}_i(\mathbf{r}) \parallel$ if it is squeezed by other surrounding cells. Followed by a preliminary study of parameters, we set,
  $$\begin{aligned} {\lambda _\mathrm{d}} = {\left\{ \begin{array}{ll} 10\ \mathrm{min}^{-1}, &{}\mathrm{if} \parallel \hat{M}_i(\mathbf{r}) \parallel < 0.03 \quad \frac{\mathrm{kg\,\cdot }\, \upmu \mathrm{m}}{\mathrm{min}^2}\\ 0 \ \mathrm{min}^{-1}, &{}\mathrm{if} \parallel \hat{M}_i(\mathbf{r}) \parallel \geqslant 0.03 \quad \frac{\mathrm{kg\, \cdot }\, \upmu \mathrm{m}}{\mathrm{min}^2}\\ &{} \qquad \quad \mathrm{for }\ \mathrm{epithelial} \ \mathrm{cells} \end{array}\right. } \end{aligned}$$
  $$\begin{aligned} {\lambda _\mathrm{d}} = {\left\{ \begin{array}{ll} 10\ \mathrm{min}^{-1}, &{}\mathrm{if} \parallel \hat{M}_i(\mathbf{r}) \parallel < 0.04 \quad \frac{\mathrm{kg\,\cdot }\, \upmu \mathrm{m}}{\mathrm{min}^2}\\ 0\ \mathrm{min}^{-1}, &{}\mathrm{if} \parallel \hat{M}_i(\mathbf{r}) \parallel \geqslant 0.04 \quad \frac{\mathrm{kg\,\cdot }\, \upmu \mathrm{m}}{\mathrm{min}^2}\\ &{} \qquad \qquad \mathrm{for} \ \mathrm{cancer} \ \mathrm{cells} \end{array}\right. } \end{aligned}$$
  $$\begin{aligned} {\lambda _\mathrm{a}} = {\left\{ \begin{array}{ll} 10\ \mathrm{min}^{-1}, &{}\mathrm{if} \parallel \hat{M}_i(\mathbf{r}) \parallel \geqslant 0.1 \quad \ \frac{\mathrm{kg\,\cdot }\, \upmu \mathrm{m}}{\mathrm{min}^2}\\ 0\ \mathrm{min}^{-1}, &{}\mathrm{if} \parallel \hat{M}_i(\mathbf{r}) \parallel < 0.1 \quad \ \frac{\mathrm{kg\,\cdot }\, \upmu \mathrm{m}}{\mathrm{min}^2}\\ &{} \qquad \quad \mathrm{for} \ \mathrm{epithelial} \ \mathrm{cells} \end{array}\right. } \end{aligned}$$
  26 $$\begin{aligned} {\lambda _\mathrm{m}} = {\left\{ \begin{array}{ll} 10\ \mathrm{min}^{-1}, &{}if \parallel \hat{M}_i(\mathbf{r}) \parallel \geqslant 0.05 \quad \frac{\mathrm{kg\,\cdot } \,\upmu \mathrm{m}}{\mathrm{min}^2}\\ 0\ \mathrm{min}^{-1}, &{}if \parallel \hat{M}_i(\mathbf{r}) \parallel < 0.05 \quad \frac{\mathrm{kg\,\cdot }\, \upmu \mathrm{m}}{\mathrm{min}^2}\\ &{} \qquad \quad \mathrm{for} \ \mathrm{epithelial} \ \mathrm{cells} \end{array}\right. } \end{aligned}$$
  Here, the corresponding probability is around 0.6321 by Eq. (23) within a time interval of $\Delta t = 0.1$ min if the probability rate is 10 $\mathrm{min}^{-1}$.
- Secondly, we assume that there is a period of time, in which a new cell grows. The length of this period is referred to as the growth time. After growth, the cell is able to
  - divide, if its growth time $\tau _\mathrm{d}$ exceeds 5 min, that is $\tau _\mathrm{d} \ge 5$ min;
  - mutate, if its growth time $\tau _\mathrm{m}$ exceeds 10 min, $\tau _\mathrm{m} \ge 10$ min;
  - apoptosis, if its growth time $\tau _\mathrm{a}$ exceeds 10 min, $\tau _\mathrm{a} \ge 10$ min.

Cells are allowed to slightly overlap other cells obtaining a repelling force and then repel each other and move away. Moreover, the repelling force increases significantly as the overlap distance goes up. In other words, cell contact inhibition impedes the cell division probability rate. This is also demonstrated by Nelson and Chen (2002) and Chen et al. (1997) who show that inhibition of cell division follows the reduction in cell area by mechanical constraint. To make the problem tractable, we only consider the change in mitotic probability rate rather than the change of cell area. The $\lambda _d$ equals 10 min$^{-1}$ after a time interval $\tau _d = 5$ min and drops from 10 to 0 min$^{-1}$ if the mechanical force is sufficiently large which is $\parallel \hat{M}_i(\mathbf{r}) \parallel \, \geqslant \,$ 0.03 kg $\cdot \,\upmu$m/min$^2$. Malumbres and Barbacid (2001) report that the tumor cells have proliferative advantage due to increased mitogenic signaling and/or the lower threshold required for cell-cycle commitment. Therefore, the threshold of strain energy density for $\lambda _d$ of cancer cells is slightly changed to 0.04 kg $\cdot \,\upmu$m/min$^2$ based on the findings by Malumbres and Barbacid (2001). One cell can divide into two cells, and the daughter cell moves away from the mother cell gradually because of the invagination force, to reach an equilibrium state for their separation distance. Moreover, normal cells exhibit aging, with a limited maximum times of division, such as a human somatic cell can divide approximately 50–100 times in culture (Harley et al. 1994). In contrast, most cancer cells do not possess a maximum number of division times, which leads to ‘immortal’ cells with ‘infinite’ division chains.

Many epithelial cells are subject to cell substrate contact-dependent proliferation and a loss of cell substrate contact is able to trigger a kind of selective programmed cell apoptosis, called *anoikis* (Stupack and Cheresh 2002; Li et al. 2003; Warchol 2002; Klekotka et al. 2001). In the 2D simulation, we impose that the epithelial cell starts to die with the probability rate of $\lambda _\mathrm{a} = 10$ $\mathrm{min}^{-1}$ when it senses the value of contact force $\parallel \hat{M}_i(\mathbf{r}) \parallel \,\geqslant \,$0.1 kg $\cdot \,\upmu$m/min$^2$ after a growth period of $\tau _\mathrm{a} = 10$ min. In this case, one epithelial cell has been surrounded by more than one layer of six cells in a large cell density over a considerable interval of time. For cancer cells, Delarue et al. (2014) find that a compressive stress could decrease the division of carcinoma cells rather than increase apoptosis. Therefore, cancer cells are assumed to die as a result of engulfment by T cells instead of mechanical stimuli.

We also consider the condition where at times an error occurs in copying of the genes during cell division and a mutation is formed. In that case, a gene has been damaged, lost or copied twice. The changes in genes could be a result of one or more reasons from physical, chemical or biological factors that were mentioned in the introduction. According to Farge (2003), many developmental genes of embryo cells are regulated by mechanical force. Kumar and Weaver (2009) report that the balance of mechanical forces, which originate from neighboring cells or the ECM, can regulate a surprisingly wide range of cellular properties that are all critical to tumorigenesis, including structure, motility, proliferation and differentiation. For the principles of how cell senses mechanical signals and convert them into changes in cellular biochemistry, one can refer to Huang and Ingber (2005) which unites cellular mechanotransduction with oncogenic signaling. Regarding to the breast cancer research in the work by Paszek and Weaver (2004), tensional force plays a potentially important role in mammary gland development and tumorigenesis. On a molecular level, compression stress is able to alter the behavior of normal cells by influencing the impact of some chemokines. Furthermore, compression stress is able to alter the behavior of transformed mammary epithelial cells by changing gene and protein expression. Hence, the mechanical signal implicitly influences the mutation rate of epithelial cells to cancer cells. Since the mechanical signals are dealt with using the strain energy density, we hypothesize that the cell mutation with the probability rate of $\lambda _\mathrm{m} = 10$ $\mathrm{min}^{-1}$ is only affected by a large strain energy density; hencewith, the cells are allowed to mutate to cancer cells if $\parallel \hat{M}_i(\mathbf{r}) \parallel \,\geqslant \,$ 0.05 kg $\cdot \,\upmu$m/min$^2$ after the time interval $\tau _\mathrm{m} = 10$ min.

### The migration of T cells in the non-isotropy collagen network

In this study, the formalism by Cumming et al. (2010) is used to describe the structure of the collagen and fibrin. To this extent, an orientation tensor $\varPsi (t,\mathbf{x})$ is introduced, where *t* and $\mathbf{x}$, respectively, denote time and position in space. In the two-dimensional setting, the entries of the symmetric tensor $\varPsi$ are arranged by its spectral decomposition:

27 $$\begin{aligned} \varPsi (t,\mathbf{x}) = \begin{pmatrix} \varPsi _{xx} &{} \varPsi _{xy} \\ \varPsi _{xy} &{} \varPsi _{yy} \end{pmatrix}. \end{aligned}$$

Stromal extracellular matrix (ECM) prevents T cells and drug delivery entering the tumor islets, which causes their migration around the islets oriented parallel to the stromal ECM. On the other hand, the movement of T cells is also affected by the concentration of chemokines (Salmon and Donnadieu 2012). Therefore, we suppose that T cells are able to enter the islets eventually with high concentration of a chemokine secreted by cancer cells. The orientation tensor is composed as the sum of its orthogonal and tangential products, which are coming from the chemokine and stromal components part, respectively. Thus, the orientation tensor $\varPsi$ can be represented by

28 $$\begin{aligned} \varPsi = \lambda _1 \mathbf{w_1w_1}^T+\lambda _2\mathbf{w_2w_2}^T, \end{aligned}$$

where the eigenvalues $\lambda _1$ and $\lambda _2$ represent the corresponding weights and the eigenvectors $\mathbf{w_1}$ and $\mathbf{w_2}$ are orthogonal and tangential components.

The research data from Bougherara et al. (2015) reveals that the density and orientation of collagen fibers control the distribution and migration of T cells as well as their ability to infiltrate tumor islets. Furthermore, their experiments illustrate that CD8 T cells migrate faster in a loose-collagen area and reduce its velocity once encountering an obstacle with densely distributed collagen fibers. At present, we assume that the tumor peripheral collagen fibrin has an uniform density and introduce a constant *k* that represents a measure for the amount that anisotropy contributed to migration which is also a fixed attenuation faction for orthogonal velocity of a cell, which reads as

29 $$\begin{aligned} \frac{\partial v}{\partial s} = -kv, \end{aligned}$$

where *s* is the penetration depth with respect to outer peripheral region and *v* is given by

30 $$\begin{aligned} v = v^0\mathrm{e}^{-ks}, \end{aligned}$$

here $v^0$ denotes the instantaneous velocity at which cells enter the outer boundary. This approach is in line with the formalism by Cumming et al. (2010). Therefore, the orientation tensor $\varPsi$ is improved slightly to,

31 $$\begin{aligned} \varPsi = v^0\mathrm{e}^{-ks}\lambda _1 \mathbf{w_1w_1}^T+v^0\lambda _2\mathbf{w_2w_2}^T. \end{aligned}$$

The *k* value is investigated in relation to the different density gradient of collagen and fiber in the computational framework. As the density gradient gets higher, the faster the radial velocity decays. The real ‘peritumoral zone’ contains a complicated mixture of neoplastic cells and tumor stroma which distribute in irregular strands or septa (Ruiter et al. 2002). In this case, the density of fibers is not uniform where the Wiener process should be taken into the account.

### Chemokine model

Chemokines are a class of cytokines that guide cells through chemotactic movement. They are involved in many physiological and pathological processes through combining with their corresponding receptors in cells, such as cell growth, differentiation, tumor progression, immune activities, etc.

Tumors have been observed to produce a variety of chemokines and the tumor-derived chemokines make an attractive target for tumor-reactive T cells to fight against them (Van Damme et al. 1992; Kershaw et al. 2002). Colombo and Trinchieri (2002) report that chemokine interleulin-12 (IL-12) acts on T cells and NK cells in antitumor immunity and immunotherapy. Furthermore, Kershaw et al. (2002) verify their hypothesis that T cells with receptor CXCR2 move toward a source of tumor-derived chemokine, Gro-$\alpha$. Therefore, it is assumed that only one kind of chemokine, which is a cytokine secreted by cancer cells, is able to attract the T-lymphocytes to move toward cancer cells. As the number of cancer cells increases, T cells migrate in the direction of the gradient of the chemokine. The reaction-diffusion equation (32) is used to describe the rate of change in the concentration of the chemokine as follows,

32 $$\begin{aligned} \frac{\partial c}{\partial t}- D_\mathrm{c}\nabla c = \sum _{j \in \mathbb {K}(t)}\gamma _j(t) \delta (\mathbf{r}-\mathbf{r}_j(t)), \quad j \in \mathbb {K}(t), \end{aligned}$$

where *c* and $D_\mathrm{c}$ represent the concentration and diffusion coefficient of chemokine. The $\delta (\mathbf{r})$ is Dirac delta function for each cancer cell *j* at time *t* and $\gamma _j(t)$ is the corresponding chemokine secretion rate by the cancer cells. Furthermore, $\mathbb {K}(t)$ denotes the set of active cancer cells at time *t*. By solving the steady-state counterpart of the partial differential equation, we get,

33 $$\begin{aligned} c(\mathbf{r}) =&- \sum _{j\in \mathbb {K}(t)}\frac{\gamma _j(t)}{2\pi D_\mathrm{c}}\mathrm{log}\parallel \mathbf{r}-\mathbf{r}_j(t)\parallel ,\nonumber \\&\mathrm{if} \parallel \mathbf{r}-\mathbf{r}_j(t)\parallel \le 1, \quad j \in \mathbb {K}(t). \end{aligned}$$

This expression is used to model chemotaxis of the T cells toward the cancer cells. Note that we only use the gradient of the above expression and that the relation is only phenomenological. Using a full time-dependent solution of Eq. (32) requires the storage of positions at all times. This makes the scheme expensive.

### The migration of T-lymphocytes

We describe the migration of epithelial and cancer cells based on the traction force as well as on random walk. T-lymphocyte cells migrate according to the gradient of the concentration of chemokines (Salmon and Donnadieu 2012) and collagen orientation (Bougherara et al. 2015) instead of according to traction force. Here, the displacement of T cells is expressed by

34 $$\begin{aligned} d\mathbf{r}_j(t) =&\, \varPsi \left[ \beta \nabla c(t, \mathbf{r}_j(t))\mathrm{d}t + \sqrt{2D} \mathrm{d}{} \mathbf{W}(t)\right] \nonumber \\&-\sum _{l \in \{j_1,\ldots ,j_k\}}M^{jl}\mathrm{d}t, \quad j \in \mathbb {T}(t), \end{aligned}$$

where the set $\{ j_1,\ldots ,j_k \}$ defines the set of cells that are in mechanical contact with cell *j*. Once again, $\mathrm{d}{} \mathbf{W(t)}$ is a vector Wiener process and $\beta$ and *D*, respectively, represent the chemotactic constant and diffusivity of the T-lymphocytes. The set of T-lymphocytes is represented by $\mathbb {T}(t)$.

Similarly, T-lymphocytes are not allowed to overlap too much under cell repulsive force described by the second part in Eq. (34), which describes the contribution to T cells migration as a result of invagination.

**Fig. 1:**
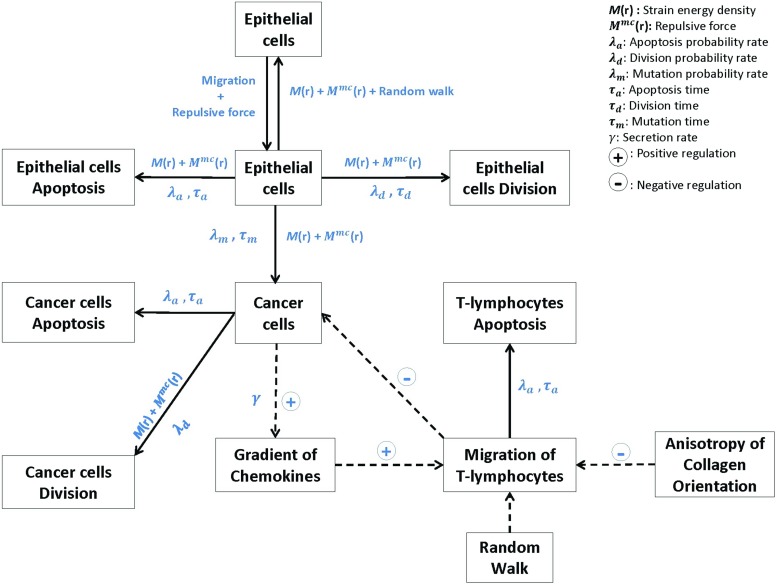
Schematic representation of the cross talk among the epithelial cells, cancer cells and immune cells in the microenvironment of a pancreatic tumor islet. The solid arrows represent the influence of a cell phenotype, where the corresponding mathematical variables have been indicated. Dotted arrows indicate the positive or negative regulations

For an overview of the cross talk among epithelial cells, cancer cells and immune cells in the microenvironment of a pancreatic tumor islet, a pictorial diagram is presented in Fig. 1 which also includes the mathematical variables and the direction of the mathematical relations.

## Numerical method

### Epithelial and cancer cells

If cells just come into mechanical contact, then the higher-order derivatives of strain energy density with respect to the intercellular distance are subject to a discontinuity. Therefore, we use the Euler–Maruyama method for time integration, which is a generalization of the ordinary forward Euler method for initial value problems to stochastic differential equations. Higher-order methods for the time integration do not seem to improve the accuracy because of the dependence between $M_{ij}$ and *h*. We evaluate the nonlinear parts at the previous time step. In this way, we circumvent the need of solving a nonlinear problem at each time step. Of course, this will induce some numerical stability criteria so that the time step cannot be chosen arbitrarily large to avoid numerical instability. The differential equation of the displacement is generally given by

35 $$\begin{aligned} d\mathbf{r}_i(t)= \alpha _i \hat{M}_i(\mathbf{r})\hat{\mathbf{z}}_i \mathrm{d}t+\sqrt{2D} \mathrm{d}{} \mathbf{W(t)}, \quad i \in \mathbb {W}(t), \end{aligned}$$

where $\alpha _i$ denotes the rate parameter mentioned in the model section, D denotes the cell diffusion coefficient and the random variable $\mathbf dW(t)$ denotes a vector Wiener process whose entries are identically distributed normal random variables with variance *dt* and expected value zero. Further, $\mathbb {W}(t)$ represents the set of epithelial cells and cancer cells. Therefore, the actual position of cell *i* at time *t* can be obtained from,

36 $$\begin{aligned} \mathbf{r}_i^t = \mathbf{r}_i^{t-1} + \Delta t \alpha _i \hat{M}_i(\mathbf{r}^t) + \sqrt{2 D} \Delta \mathbf{W}. \end{aligned}$$

Here, $\Delta \mathbf{W}$ represents a two-dimensional vector with independent stochastic variables from a normal distribution with zero mean and variance $\Delta t$.

Since the cells may collide into one another, they should not overlap each other totally. Therefore, we require their displacement to be less than one-fourth of their diameter. This criterion is quantified by

37 $$\begin{aligned} \parallel \mathbf{r}_i^t - \mathbf{r}_i^{t-1} \parallel \,=\, \mathrm{max}\parallel \mathbf{v}_i\parallel \Delta t \le \frac{R}{2}, \end{aligned}$$

where *R* is the radius of epithelial or cancer cells and $\mathbf{v}_i$ is the equilibrium velocity of cell *i*. From equation (37), the time step is determined by

38 $$\begin{aligned} \Delta t = \mathrm{min}\left( 0.1, \ \frac{R}{2 \mathrm{max}\parallel \mathbf{v}_i\parallel }\right) , \end{aligned}$$

here we use a default value 0.1 min for time step. Whereas if the migration speed of the cells is large, then the time step is adjusted to $\Delta t = \frac{R}{2 \mathrm{max}\parallel \mathbf{v}_i\parallel }.$ This limitation of the time step guarantees that the cells do not move too much over a time interval and do not entirely coincide with each other. Furthermore, numerical experiments indicate that numerical stability is also guaranteed if the above criterion in Eq. (38) is satisfied. This issue deserves some further numerical consideration in mathematical rigor.

**Table 1:** Parameter values

Parameter	Meaning	Value	Unit	Source
*R*	Radius of epithelial and cancer cell	2.5	$\upmu$m	Dudaie et al. 2015
$R_\mathrm{t}$	Radius of T cells	2	$\upmu$m	Dudaie et al. 2015
*F*	Cell traction force	(10–25)$\cdot 10^{2}$	kg $\cdot \,\upmu$m/min$^{2}$	Reinhart-King et al. (2003) and Ganz et al. (2006)
$$E_\mathrm{s}$$	Substrate elasticity	5$$\cdot 10^{-5}$$	kg/($$\upmu $$m $$\cdot $$ min$$^{2})$$	Dudaie et al. (2015)
$$E_\mathrm{c}$$	Cell elasticity	0.5$$\cdot 10^{-5}$$	kg/($$\upmu $$m $$\cdot $$ min$$^{2})$$	Dudaie et al. (2015)
$\beta$	Cell mobility coefficient	1	min$^{-1}$	Vermolen and Gefen (2012)
$\mu$	Friction coefficient	0.2	-	Dudaie et al. (2015)
*D*	Cell diffusivity	0.005	$\upmu$m/min	Estimated in this study
$D_\mathrm{c}$	Coefficient of chemokine diffusivity	0.001	$\upmu$m/min	Estimated in this study
$\gamma$	Secretion rate of the chemokine	10	min$^{-1}$	Estimated in this study

**Fig. 2:**
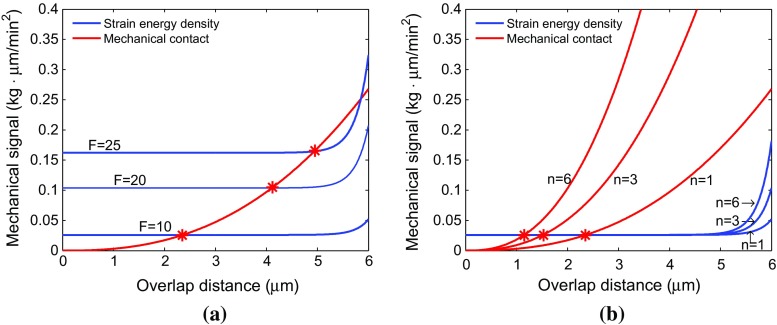
Red and blue lines represent mechanical contact force and strain energy density, respectively. **a** Compares the strain energy density with different cell traction force *F* values. **b** Compares the mechanical contact force and strain energy density of one cell when it is surrounded by other one, three and six cells, respectively, for *F* = 10 kg $\cdot \,\upmu$m/min$^{2}$

### T-lymphocytes

We use the same Euler–Maruyama method for T cells migration. The displacement of T cells is chemotaxis, and we also incorporate the random walk to form stochastic differential equations. With the explained parameters in the former section, we calculate the actual positon of T cells *j* at time *t* by

39 $$\begin{aligned} \mathbf{r}_j^t&= \mathbf{r}_j^{t-1}+\varPsi \left[ \nabla c(t, \mathbf{x}_j^{t-1})\Delta t + \sqrt{2D} \Delta \mathbf{W}\right] -\nonumber \\&\sum _{l \in \{l_1,\ldots ,l_k\}}M^{jl}\Delta t \quad j \in \mathbb {T}(t), \end{aligned}$$

here the $M^{jl}$ represents repulsive force between a T cells *j* and a cancer cell *l*.

With cell contact inhibition, T cells will be bounced once they collide with each other. Therefore, we suppose that the fourth of their diameter is the maximum overlapping distance. We use the same criterion in Eq. (38).

Furthermore, the same method is used to deal with the collision of T cells and epithelial as well as cancer cells. Since T cells are smaller than the other cells, we suppose that the maximum overlapping distance of them depends on the radius of the T cells.

## The numerical simulations

### Parameter values

To mimic tumor initiation and the T cells-mediated immune response in pancreatic tumor islets as well as possible, we chose the parameter values based on available sources in literature as much as possible. For those cases where literature values are not readily available, we make educational guesses based on the expected behavior. Table 1 lists all parameters values.

### Results

For the two-dimensional simulation, the projection of each cell is defined as a circle on the substrate and a large circular domain with a radius of 35 $\upmu$m is used to simulate a tumor islet. Regarding cancerous mutation, in the simulations, we highlight the mutation by a change of color from blue to red in the figures. In order to predict the impact of the non-isotropic fibrin network in different immune responses mediated by T-lymphocytes, we simulate tumor islets under different conditions for immunity with stromal ECM orientation and without stromal ECM orientation.

First of all, we investigate the changes in strain energy density as well as mechanical contact force in different situations with respect to the overlap distance in Fig. 2. The result in Fig. 2a shows that the equilibrium overlap distance coming from strain energy density and mechanical repulsive force for two cells increases as the *F* value arises and the best reasonable choice for *F* is 10 kg $$\cdot \,\upmu $$m/min$$^{2} $$ considering the cell radius. Moreover, in Fig. 2b, the curves of mechanical contact force vary a lot when one cell is surrounded by other one, three and six cells and the maximum equilibrium overlap distance is approximately 2.35 $$\upmu $$m when one cell is contacted by another one. This amount of overlap is deemed acceptable.

**Fig. 3:**
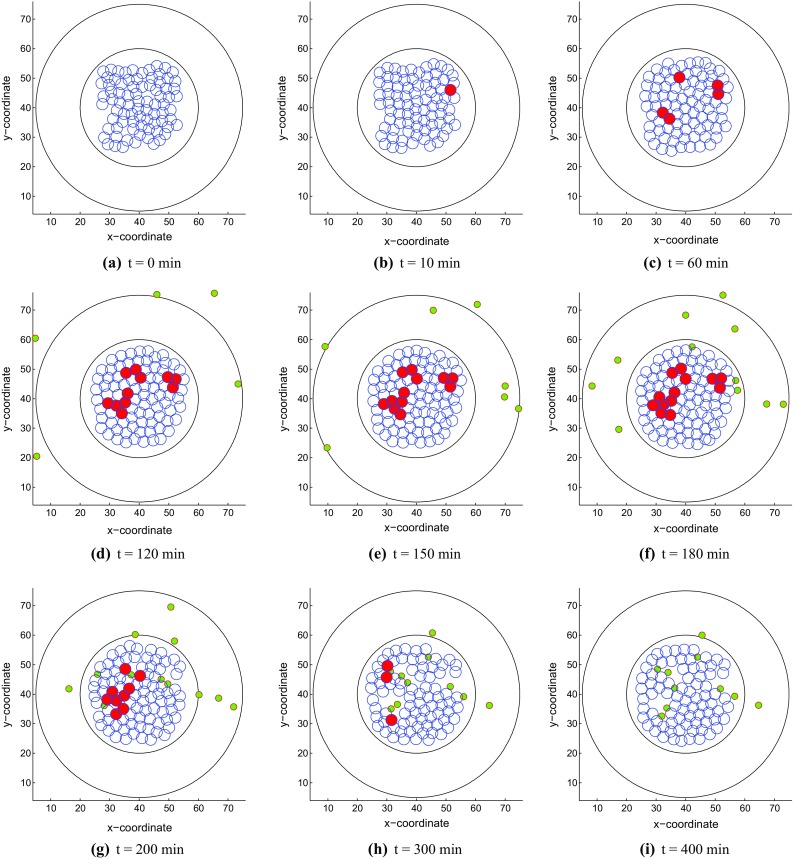
Snapshots of the tumor islet without anisotropic collagen orientation ($k = 0$) under a strong immune reaction. The blue, red and green circles denote the epithelial, cancer and T-lymphocytes, respectively. **a** *t* = 0 min, **b** *t* = 10 min, **c** *t* = 60 min, **d** *t* = 120 min, **e** *t* = 150 min, **f** *t* = 180 min, **g** *t* = 200 min, **h** *t* = 300 min and **i** *t* = 400 min

#### Tumor islets without stromal ECM orientation

Firstly, we consider the simulation of tumor islets without any anisotropic collagen orientation, which means $k = 0$ in Eq. (31); hence, the migration of T cells is determined by the concentration gradient of chemokine.

**Fig. 4:**
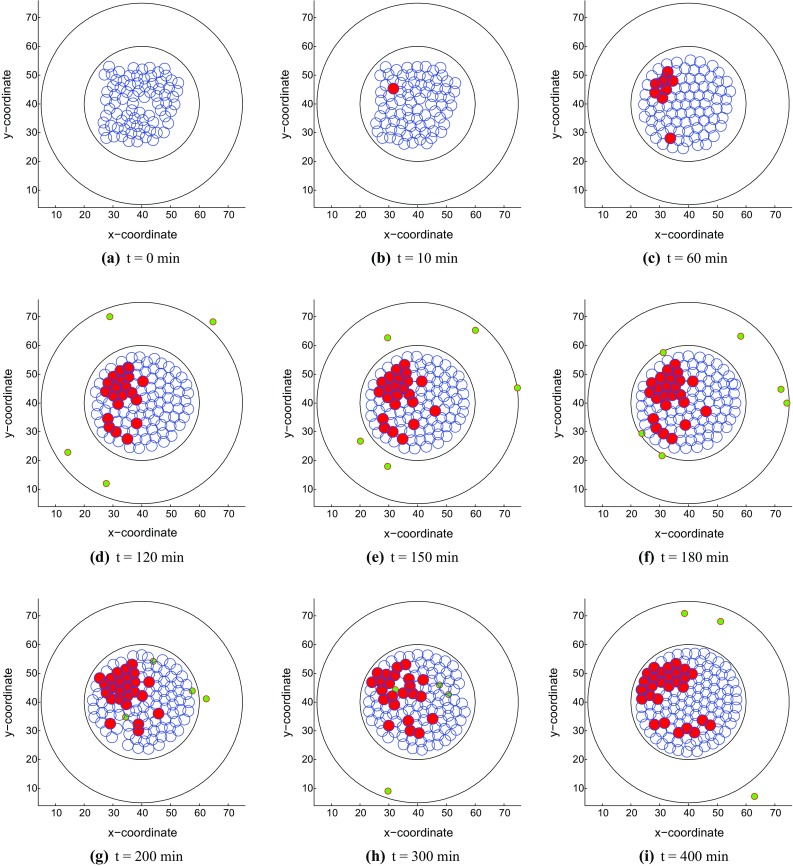
Snapshots of the tumor islet without anisotropic collagen orientation ($k = 0$) under a weak immune reaction. The blue, red and green circles denote the epithelial, cancer and T-lymphocytes, respectively. **a** *t* = 0 min, **b** *t* = 10 min, **c** *t* = 60 min, **d** *t* = 120 min, **e** *t* = 150 min, **f** *t* = 180 min, **g** *t* = 200 min, **h** *t* = 300 min and **i** *t* = 400 min

With a large mechanical stimulus, an epithelial cell mutates to a tumor cell in Fig. 3b and it starts to divide subsequently. Since the tumor cells release a chemokine, T cells move to the islet from different directions according to the chemokine signal. Cancer cells may be engulfed by T cells when they are in contact for some time, concurrently the T cells also have a certain probability of death. In this model, T cells and epithelial cells are not allowed to physically overlap as a result of mechanical interaction. Here, we simulate two kinds of results with different immune responses as follows: (1) Figure 3 shows the tumor islet with a strong immune response in which T-lymphocytes win eventually; (2) Figure 4 describes the tumor islet with a weak immune response so that the tumor colony occupies the entire region in the course of time. Note that for the differences between strong and weak immune systems, we describe the strong and weak immune systems as follows

- $N_\mathrm{s} = 2N_\mathrm{w}$, here $N_\mathrm{s}$ and $N_\mathrm{w}$ denote the number of T cells in the strong immune system and weak immune system, respectively.
- A T cells has a probability rate $\lambda _\mathrm{d} = 10$ to die if the distance between the T cells and cancerous cell satisfies $\parallel \mathbf{r}_t - \mathbf{r}_c\parallel \le 2.5 \ \upmu$m over a time interval $\tau =10$ min in weak immune system. Whereas death of T cells with the same probability rate $\lambda _\mathrm{d} = 10$ sets in if $\parallel \mathbf{r}_t - \mathbf{r}_c\parallel \le 3.5 \ \upmu$m over a time interval $\tau =5$ min in the strong immune system.

**Fig. 5:**
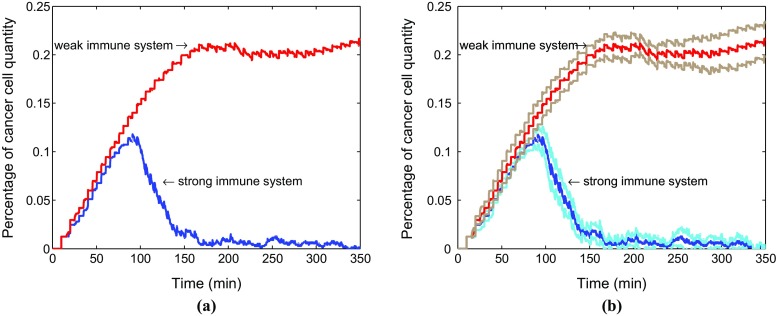
Comparison for change of the percentage of cancer cells in total cells in two situations. The red and blue lines represent the evolution of the percentage of cancer cells in a weak immune system and a strong immune system, respectively (**a**). Furthermore, the corresponding confidence intervals are shown in **b**. The brown and light blue lines denote the confidence intervals of a weak immune system as well as a strong immune system in the tumor islets without anisotropic collagen orientation ($k = 0$), respectively

Figure 5 compares the evolution of the percentage of the cancer cells in total cells as a function of time in two situations. The number percent of cancer cells accounts for a large advantage in a weak immune system compared with the strong immune system. In order to find a confidence interval with 95% confidence level, a sample for 10 runs has been chosen and results are shown in Fig. 5. Figure 5a describes the average results and Fig. 5b shows the average results with corresponding confidence intervals. The weak immune system fails to control the cancer cells although a small reduction appears around $\tau =$ 250 min.

#### Tumor islets with stromal ECM orientation

Subsequently, we incorporate the anisotropic collagen orientation into this model and the 15-$\upmu$m-thick annular gray region visualizes the extracellular matrix with rich fibers of collagen and myofibroblasts. The function contribution of desmoplastic stroma is unknown and controversial; however, it is reported to have a suppressive role for the immune response (Rhim et al. 2014; Salmon and Donnadieu 2012). Herein, we model this phenomenon in order to provide some ideas for further research.

**Fig. 6:**
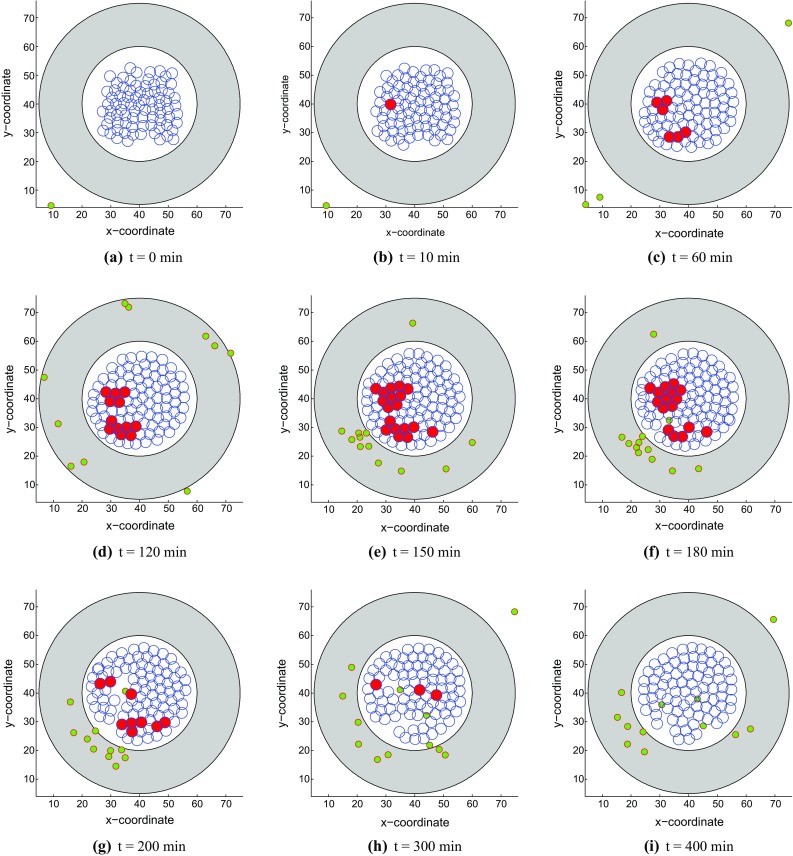
Snapshots of the tumor islet with anisotropic collagen orientation ($k = 0.3$) under a strong immune reaction. The blue, red and green circles denote the epithelial, cancer and T-lymphocytes, respectively. **a** *t* = 0 min, **b** *t* = 10 min, **c** *t* = 60 min, **d** *t* = 120 min, **e** *t* = 150 min, **f** *t*= 180 min, **g** *t* = 200 min, **h** *t* = 300 min, **i** *t* = 400 min.

With the same parameters, the epithelial cells are allowed to mutate to cancer cells (Fig. 6b), which can arouse the immune T cells chemotaxis (Fig. 6d). After T cells enter into the stroma, they sense the local orientation as well as chemokine signal and move to the place where normally gathered many cancer cells, see Fig. 6e. The reason why T cells move along the collagen is because the anisotropic fibers are positioned parallel to the tumor islet boundary. Normally, tissue has isotropic fibers which has a ‘basket weave’ pattern with a fairly random orientation, whereas this stromal layer is described with more aligned collagen fibers. By experiment observation, Bougherara et al. (2015) report that T cells follow precisely the pre-defined collagen scaffold and move between two fibers. Furthermore, compared with the dense region, the loose-collagen areas have more CD8 T cells. Therefore, the T cells migration is guided by collagen orientation and affected by collagen density, however, here we suppose T cells suffer some impediment in parallel collagen fibers with uniform density and get through the barrier eventually with the increase of the number of cancer cells.

**Fig. 7:**
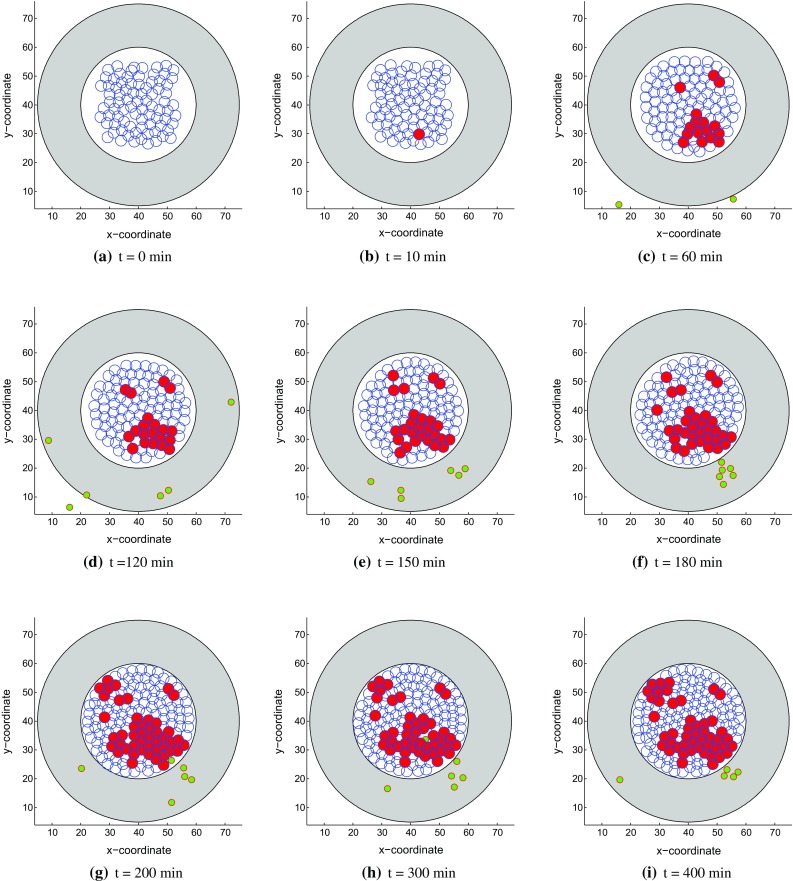
Snapshots of the tumor islet with anisotropic collagen orientation ($k = 0.3$) under a weak immune reaction. The blue, red and green circles denote the epithelial, cancer and T-lymphocytes, respectively. **a** *t* = 0 min, **b** *t* = 10 min, **c** *t* = 60 min, **d** *t* = 120 min, **e** *t* = 150 min, **f** *t* = 180 min, **g** *t* = 200 min, **h** *t* = 300 min and **i** *t* = 400 min

In this part, we also simulate two kinds of results. T cells eliminate cancer cells and reach a dynamic equilibrium with a strong immune reaction in Fig. 6 while cancer cells proliferate out of control in a weak immune system in Fig. 7.

**Fig. 8:**
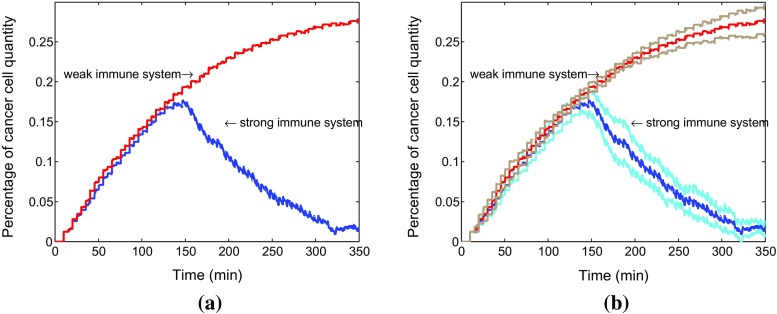
**a** Comparison for change of the percentage of cancer cells in total cells in two situations. The red and blue lines represent the cancer cell percentage change in a weak immune system and a strong immune system, respectively. Furthermore, the corresponding confidence intervals are shown in **b**. The brown and light blue lines denote the confidence intervals of a weak immune system as well as a strong immune system in the tumor islets with anisotropic collagen orientation ($k = 0.3$), respectively

**Fig. 9:**
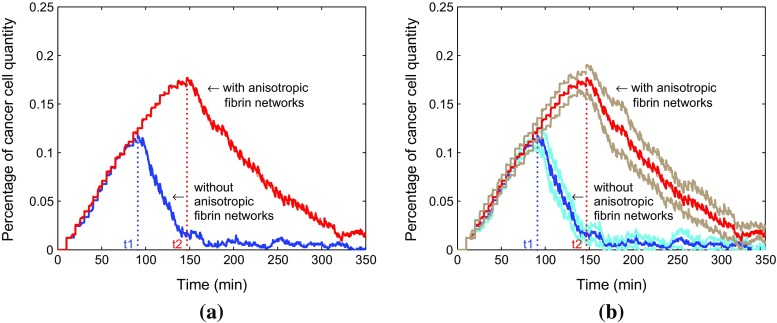
**a** Comparison for time to fight the cancer cells. The blue and red lines represent the strong immune system without anisotropic collagen orientation (with the response time $t_1$) and with anisotropic collagen orientation (with the response time $t_2$), respectively. Furthermore, the corresponding confidence intervals are shown in **b**. The light blue and brown lines denote the confidence intervals of strong immune systems in the tumor islets without anisotropic collagen orientation ($k = 0$) as well as with anisotropic collagen orientation ($k= 0.3$), respectively

Similarly, the percentage of cancer cells quantity in both situations is compared over time in Fig. 8 by using average data coming from a sample of 10 runs with 95% confidence level. This amounts to running ten simulations where the parameters are taken randomly using the normal distribution. The 95% interval of confidence is subsequently computed via $$\left( \overline{x}-1.96*\frac{\sigma }{\sqrt{n}}, \ \overline{x}+1.96*\frac{\sigma }{\sqrt{n}}\right) $$. After a while, the fraction number of cancer cells is apparently bigger in a weak immune system. Furthermore, we compare the time responses with corresponding 95% confidence intervals in both strong immune system with collagen and without collagen (see Fig. 9b). The figure shows that the anisotropic collagen contributes a lot with $$k > 0 $$ for migration of T cells in blue line, which describes the strong immune response. Therefore, T cells need more time $$t_2$$ to get the cancer cells in the tumor islet with collagen, and the corresponding number of cancer cells reaches a higher level. Therefore, stromal collagen impedes the immune response of T cells.

**Fig. 10:**
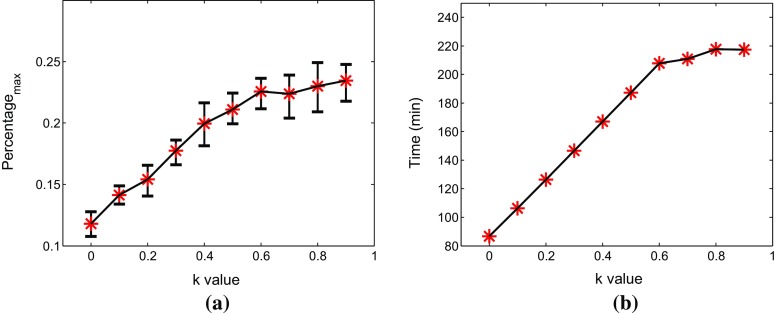
**a** Relation between the maximum percentage of number of cancer cells and the *k* value in the tumor islet with anisotropic collagen orientation. The red asterisks and black lines represent average date and $95\%$ confidence intervals. **b** The relation between the time of T cells to fight the cancer cells with and the *k* value

**Fig. 11:**
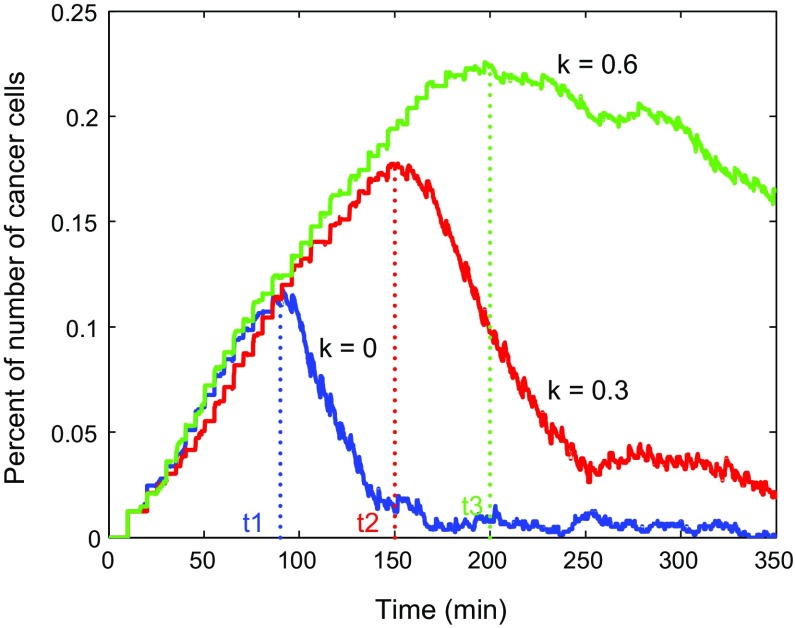
Evolution of percentage of number of cancer cells and immune time of T cells with respect to *k* = 0, 0.3, 0.6, respectively

Differences in collagen fiber density lead to different orientation effects. In order to further investigate the effect of tumor surrounding collagen on the T cells movement in varying degrees, we give ten different values to *k* from 0 to 0.9. As we expected, the inhibitory effect of collagen increases with the increase of *k* value. This inhibitory is presented mainly by the parallel aligned fiber barrier on the attenuation of the cell radial velocity and orientation on the tangential direction. Correspondingly, the immune response time of T cells and maximum percentage of number of cancer cells are two important criteria for judging the inhibitory effect, which are compared in Fig. 10. The mean of the data for different *k* values is represented by red asterisks coming from a sample of 10 runs. Both the immune time and maximum number monotonically increase significantly at the beginning and then they gradually stabilize. Figure 11 shows the evolution of the number of cancer cells with respect to time for $k =$ 0, 0.3, 0.6, respectively.

In conclusion, specific T cells-mediated immunity plays an essential role in tumor islets progression. Pathologists have found that almost each individual has cancer cells after a large number of autopsy and pathological examination. However, most individuals only have a very few cancer cells in vivo without any symptoms, which are not able to form cancer. Few cancer cells only can be seen under a microscope by a biopsy so that it is difficult to be diagnosed. Therefore, the immune-related theoretical principles and tumor microenvironment need to be further simulated and researched.

## Discussion

Cancer cells differ from normal cells with some characteristics, such as unlimited growth, conversion as well as metastasis. Most individuals have a good balance of proto-oncogene and anti-oncogene. However, this balance can be disturbed by some carcinogens. Usually, abnormal cells will be eliminated by the immune system before they become cancerous. Therefore, the immune response is very important to fight cancer. However, tumors have many strategies to suppress or escape the tumor-specific immunity.

In this paper, we phenomenologically model the tumor islets in pancreatic cancer, which uses the stroma to impede the immunity to some extent. As far as we know, this is the first mathematical modeling study devoted to the simulation of pancreatic cancer that takes into account orientation of the surrounding collagen. In order to predict this influence, we have three characteristics for the comparative simulation study: (1) T-lymphocytes migrate to the cancer cells without stromal ECM orientation in a strong as well as a weak immune response. It means T cells sense the chemokine signal only and move according to the concentration gradient; (2) stromal ECM orientation combined with chemokine factor guide the movement of T cells in two kinds of situations; (3) a parameter study of *k* value. Currently, we have three results listed as follows:

- The model quantifies the delay of invasion of T cells into the cancer-effected area as a result of anisotropic collagen orientation, and hence, it quantifies the increase in time to battle the cancer cells;
- The model predicts unlimited proliferation of carcinoma cells if the immune system is weak, and a state of equilibrium where cancer cells are eliminated if the immune system is sufficiently strong;
- As we expected, the obstructing effect of stromal ECM increases with the increase of *k* value which is used to denote a measure for the amount that anisotropy contributed to T cells migration. Hence, the number of cancer cells is allowed to grow to larger values if the stromal ECM around the tumor islet gets more woven parallel to the circumference of the islet.

Although this model presents the first description of cancer development in the pancreatic under the influence of orientation of the surrounding collagen, and although the modeling looks sensible and meaningful in a qualitative context, many details of cancer cells are ignored in order to get a simple, well-tractable model for this preliminary study. The following items regarding improvement of the model can be discussed:

- *Developing 3D model* The computational framework that we currently present is in a two-dimensional framework. This large simplification has been carried out to save CPU time. The main objective of this paper is to set up a formalism for the inhibition of the immune system as a consequence of the orientation of the stromal layer. However, a 3D model is more physiological to simulate the real biomedical phenomenon. Although our work is only in 2D, the main conclusions remain the same. That is, the orientation of the stromal layer poses a delay to T cells to entering the tumor islet. Therewith, the organism will have some difficulty in fighting cancer. Furthermore, the conclusions regarding the impact of the strength of the immune system on fighting cancer will be the same regardless of the dimensionality of the model. From a qualitative point, no significant changes are expected regarding dimensionality. However, if it comes to quantitative claims, then the dimensionality will have considerable impact. Therefore, in the future, we plan to develop a 3D model in a parallel computing environment.
- *Improving the probability for cell division and death* In some studies, it has been found that the length of telomere DNA of cells gradually shortens as the number of divisions of a cell increases. Lindsey et al. (1991) report that the telomere length of skin cells becomes shorter causing cell aging and lower division rates. This phenomenon is also observed for epithelial cells, T-lymphocytes and hematopoietic stem cells later. Allsopp et al. (1992) observe that different individuals’ fibroblasts have different abilities to proliferate and that the maximum number of divisions increases with increasing telomere length. Therefore, a dynamic probability for cell division or death could be incorporated into the modeling to simulate initiation of cancer through an enhanced mutation rate of individual cells. In the current model, cells divide or die depending on the strain energy density as well as fixed probability rates after some time periods; however, in future work we plan to incorporate this feature of the dynamic probability rates, which will be an innovation with respect to the existing literature. A way to do this could be the following: let N be the number of cell divisions, then we may set,
  40 $$\begin{aligned} {\lambda _N} - {\lambda _{N-1}} = C {\lambda _N} (1 - {\lambda _N}/ {\lambda _\infty }), \end{aligned}$$
  where *C* is a positive constant and $\lambda _N$ is the probability rate of cell division after *N* divisions per unit. Furthermore, $\lambda _\infty$ stands for the probability rate for cell division after an ‘infinite’ number of cell divisions. If $C > 0$, then the number of cell divisions increases, the probability rates of mutation, proliferation and death will gradually converge to $\lambda _\infty$, else convergence toward zero will be obtained.
- *Incorporating more chemical factors* In this study, the strain energy density as well as one chemokine are assumed to be the only factors for cell proliferation, apoptosis, mutation, etc. In reality, hormones, endostatin and other substances collectively influence the cell activity. Hence, one could incorporate oxygen content, nutrients, more chemokines, etc. This, however, would make the model less tractable.
- *Coupling with angiogenesis* Since the process of tumor growth is really complicated, it is not yet fully understood how the tumor grows. Modeling is still in its early stage without unified theoretical basis. Angiogenesis plays a crucial role in tumor growth and its spreading over different parts of the body; therefore, how to build a proper model describing the angiogenesis mechanism is going to be a complicated challenge. Innermost cancer cells of any colony are most likely to die first, since the concentrations of oxygen and nutrients are much lower than the concentrations on the rim of the tumor and furthermore, the mechanical contact force, they are exposed to, are much longer. We will take the concentrations of oxygen and nutrients into account for apoptosis. Cancer cells releasing angiogenic factor activate vascular epithelial cells and promote proliferation and migration of epithelial cells. We will simulate this part combined with tumor cell dynamics and associated immune responses in future work. The reader is referred to Bookholt et al. (2016), where the current cell-based model was extended and applied to angiogenesis.
- *Collagen degradation* With the growth of tumors, internal hypoxic cancer cells will die and dead cells could initiate the mechanisms of angiogenesis by secretion of cytokines. One of the first steps in neovascularization is degradation of membrane collagens by endothelial cells, which move along the chemotactic stimulus. Endothelial cells are able to degrade interstitial type I collagen by releasing MMPs (Kalebic et al. 1983). Therefore, we plan to incorporate this into the model to explore the efficacy of degradation of collagen in immune response as well as angiogenesis.
- *A parameter variation study* Besides all these questions, all models need input parameters, which are hard to find and which vary from individual to individual. Therefore, it is also important to carry out a probabilistic parameter variation study and try to get values from in vivo and in vitro measurements. Afterward, we could quantify the probability of tumor initiation, growth and seeding to other organs in terms of biophysical parameters, genetics and lifestyle in realistic settings and geometries.

The techniques that we used here reside on continuum models solved by the use of analytic expressions in terms of Green’s functions or approximations, as well as stochastic principles for cell proliferation, mutation, death and migration. Combination with finite-element strategies could improve the description regarding mechanics as well as more complicated reaction-transport equations for the chemokines. For cancer therapy, traditional methods are chemotherapy and radiotherapy, which aim at cancer cells. However, they inevitably cause varying degrees of damage and toxicity for the human body. Nowadays, cancer immunotherapy has some new developments that enlists the immune system to attack targeting tumors directly (Couzin-Frankel 2013). Thus, our original model and further mathematical simulation is very meaningful and important for cancer immunotherapy. Furthermore, it lays a foundation for cancer development and inhibition for smart health care.

## Electronic supplementary material

Below is the link to the electronic supplementary material.

Supplementary material 1 (docx 14 KB)

Supplementary material 2 (avi 11788 KB)

Supplementary material 3 (avi 16844 KB)

Supplementary material 4 (avi 16965 KB)

Supplementary material 5 (avi 13321 KB)
