# Supplementary material for: A model for cell migration in non-isotropic fibrin networks with an application to pancreatic tumor islets
Source: Biomech Model Mechanobiol. 2017 Oct 9;17(2):367–86. doi: 10.1007/s10237-017-0966-7 (PMC5845079; doi:10.1007/s10237-017-0966-7)
Supplement: Supplementary file 1 — Supplementary material 1 (docx 14 KB) [file 10237_2017_966_MOESM1_ESM.docx]

The supplementary files are four videos which present the T- lymphocyte migration with and without anisotropic collagen orientation in different immune systems, respectively. The blue, red and green circles are used to visualize the epithelial cells, cancer cells and T-lymphocytes. Moreover, the annular grey region denotes the stromal extracellular matrix with rich uniform fibres of anisotropic collagen and myofibroblasts. The caption of each video is listed as follows.

1. Video1

T- lymphocytes migration in an initial pancreatic tumor islet **without** anisotropic collagen orientation under a **strong** immune system

1. Video2

T- lymphocytes migration in an initial pancreatic tumor islet **without** anisotropic collagen orientation under a **weak** immune system

1. Video3

T- lymphocytes migration in an initial pancreatic tumor islet **with** anisotropic collagen orientation under a **strong** immune system

1. Video4

T- lymphocytes migration in an initial pancreatic tumor islet **with** anisotropic collagen orientation under a **weak** immune system
